# Use of *Yarrowia lipolytica* Lipase Immobilized in Cell Debris for the Production of Lipolyzed Milk Fat (LMF)

**DOI:** 10.3390/ijms19113413

**Published:** 2018-10-31

**Authors:** Jully L. Fraga, Adrian C. B. Penha, Adejanildo da S. Pereira, Kelly A. Silva, Emília Akil, Alexandre G. Torres, Priscilla F. F. Amaral

**Affiliations:** 1 Escola de Química, Universidade Federal do Rio de Janeiro, Rio de Janeiro, RJ 21941-909, Brazil; jully.lfraga@gmail.com (J.L.F.); acbp_adrian14@hotmail.com (A.C.B.P.); adejanildosp@gmail.com (A.d.S.P.); Kelly_alencar@id.uff.br (K.A.S.); 2 Laboratório de Bioquímica Nutricional e de Alimentos, Instituto de Química, Universidade Federal do Rio de Janeiro, Rio de Janeiro, RJ 21941-909, Brazil; emilia.akil@gmail.com (E.A.); torres@iq.ufrj.br (A.G.T.)

**Keywords:** fatty acids, lipase, cell debris, lipolyzed milk fat

## Abstract

Lipase immobilized on *Yarrowia lipolytica* cell debris after sonication of yeast cells (LipImDebri) was used in hydrolysis reaction as a novel strategy to produce lipolyzed milk fat (LMF). Extracellular (4732.1 U/L), intracellular (130.0 U/g), and cell debris (181.0 U/g) lipases were obtained in a 4 L bioreactor using residual frying oil as inducer in 24 h fermentation process. LipImDebri showed a good operational stability retaining 70% of lipolytic activity after the second cycle and 40% after the fourth. The highest degree of hydrolysis (28%) was obtained with 500 mg LipImDebri for 6 h of lipolysis of anhydrous milk fat. LMF produced with LipImDebri presented high contents of oleic (35.2%), palmitic (25.0%), and stearic (15.4%) acids and considerable amounts of odor-active short and medium chain fatty acids (C:4–C:10) (8.13%).

## 1. Introduction

For many years enzymes have been used in biotechnological processes as catalysts in several industrial sectors, mainly in food production [1]. The use of enzymes presents advantages in comparison to chemical catalysts such as mild reaction conditions and versatility [2]. Lipases (glycerol ester hydrolases E.C. 3.1.1.3.) have emerged as one of the leading biocatalysts because of the possibility to catalyze ester bond hydrolysis reactions of triacylglycerols as well as synthesis reactions, and also for their ability towards extremes of temperature, pH, and organic solvents, and chemo-, region-, and enantioselectivity [3]. Most lipases exhibit a catalytic mechanism that differentiates them from standard esterases, by the presence of two different conformations in equilibrium. A closed form (inactive) is observed when no hydrophobic surface is available and is characterized by a “lid” of helicoidal polypeptide chain that closes the catalytic site, making it inaccessible to the reaction medium. The open form (active), where this lid is entirely displaced, occurs in the presence of hydrophobic surfaces [4].

Despite the excellent reaction conditions and properties, lipases usually have to be improved before their implementation at industrial scale (where many cycles of high yield processes are desired) [5]. Immobilization improves enzyme stability, activity, reduces the inhibition by reaction products, enables continuous production, and also allows biocatalyst removal from product stream [5,6,7]. Although there are many researchers reporting enzyme immobilization techniques, only a few have indeed been commercialized because immobilization costs are usually very high [6]. This cost is generally related to the support chosen, that can be organic, inorganic, or hybrid and composite materials [8]. Methods for immobilization are divided into three categories: adsorption on a carrier (support), encapsulation in a carrier, and cross-linking (carrier-free) [9]. These categories comprise several protocols and frequently for isolated enzymes the methods based on chemical interactions are generally preferred and physical entrapment is the option for whole cell immobilization [10]. Bacteria, yeast, and fungi have been used as whole-cell biocatalysts in order to improve the cost-effectiveness of the bioconversion process [11]. *Rhizomucor miehei* and *Yarrowia lipolytica* displaying high intracellular lipase activity were immobilized in polyurethane foam, which increased lipase activity in relation to free biomass [12].

*Yarrowia lipolytica* is one of the most-studied nonconventional yeasts with special interest to biotechnological processes due to its ability to secret several metabolites in large amounts (e.g., citric acid and extracellular proteins) [13]. *Y. lipolytica*, in addition to secreting extracellular lipases, maintains a fraction of it bounded to the cell wall [14,15]. *Y. lipolytica* cell wall-bound lipases can be used as non-artificially immobilized enzymes, reducing process costs (no additional support is needed nor an immobilization process that is time consuming) and promoting an efficient hydrolysis of triacylglycerols [16]. As an immobilized biocatalyst it can also be recovered and reused, thus enabling its cost-effective use in, for example, continuous, fixed-bed operation [9].

Lipolyzed milk fat (LMF) is an important ingredient in the food industry and can be applied as additive in bakery products (breads, cakes, cookie mixes, candies, chocolate products, and toffees), and dairy products. When milk fat is used as a substrate under specific enzymatic conditions, the LMF produced may be used as a vehicle for cheese flavors [17]. Lipases obtained from different microorganisms can produce a wide range of flavors by enzyme mediated transformation. Regado et al. [18] used different commercial lipases and one cutinase to obtain cheesy flavors and observed that the free short-to-medium chain fatty acids profile formed depended on lipase’s microbial source.

In this research, lipase naturally immobilized in cell debris (bound to the cell wall) was produced during culture with a food residue, residual frying oil (RFO), of a wild type yeast isolated from Baía de Guanabara (Rio de Janeiro, Brazil), *Yarrowia lipolytica* IMUFRJ 50682. This enzyme was used to hydrolyze milk fat in order to obtain LMF flavor.

## 2. Results and Discussion

### 2.1. Production of Lipase from Y. lipolytica Immobilized in Cell Debris Induced by Residual Frying Oil

**Figure 1:**
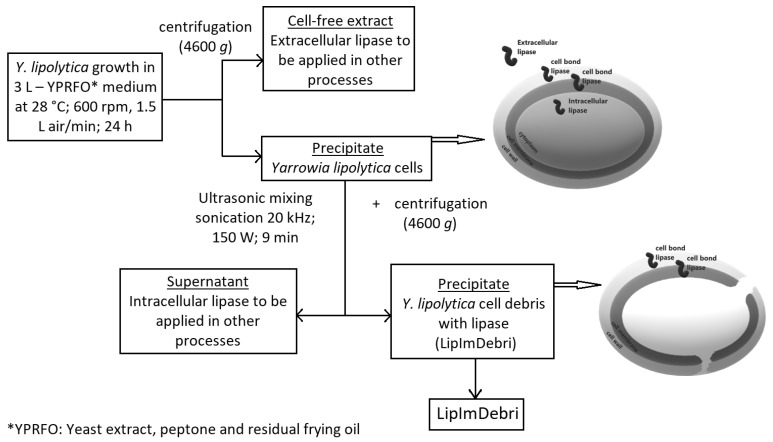
Schematic diagram of *Yarrowia lipolytica* lipase immobilized on cell debris (LipImDebri) production.

Residual frying oil (2.5% *v*/*v*) was used as an inductor for lipase production by *Y. lipolytica* in a 4 L bioreactor. Cell-free medium, after 24 h fermentation, contained approximately 4700 U/L of extracellular lipase activity. The extracellular lipase extract was used for other studies [19]. At this moment 5.7 g d.w. (dry weigh) cells/L was detected in the culture medium, resulting in, approximately 17 g d.w. of cells. The biomass of cells, generated at this same time, was sonicated and subsequently centrifuged, producing a supernatant with 130 U/g d.w. of intracellular lipase and a precipitate of cell debris with 180 U/g d.w. of lipase. This sonicated biomass was used afterwards as lipase immobilized in cell debris (LipImDebri). The schematic diagram of LipImDebri production can be visualized in Figure 1. Nunes et al. [15] had already shown the potential of using residual frying soybean oil for lipase production in shaker flasks. In the present work, the production in a bioreactor brings closer the potential to scale-up production in the future. Despite the potential presence of oil degradation products (polar triacylglycerols (TAG), di- and mono-acylglycerols, free fatty acids (FFA), oxidized FFA, among other compounds) in these residues due to long oil heating [20], lipase production by *Y. lipolytica* did not seem to be negatively affected, indicating a possible way of reducing this residue discard in the environment and reducing costs for the production of this enzyme.

### 2.2. Characterization of Lipase Immobilized in Cell Debris

**Figure 2:**
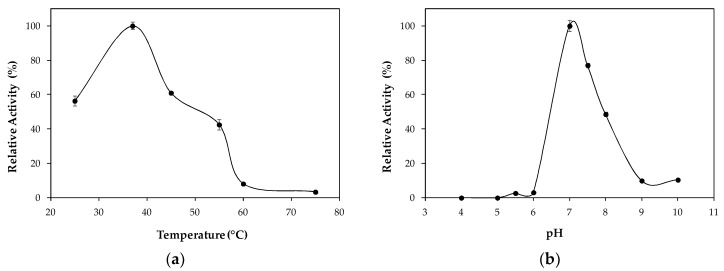
Effect of (**a**) temperature and (**b**) pH on hydrolytic activity of *Yarrowia lipolytica* lipase immobilized on cell debris (LipImDebri) in *p*-nitrophenyl laurate. Lines between dots do not represent experimental data.

The application of a biocatalyst is closely related to its optimal temperature and pH [6]. Therefore, hydrolytic activity of LipImDebri was assayed in the range of 25 to 75 °C at pH 7.0 and at different pH ranging from 4.0 to 10.0 at 37 °C, as shown in Figure 2.

The best temperature and pH for LipImDebri hydrolytic activity was 37 °C and pH 7.0 (Figure 2). Most yeast lipases have optimum temperature in the range of 30 to 40 °C [21,22,23], especially for *Y. lipolytica* [24]. Neutral pH is common for lipases [23,24], but sometimes, basic pH is observed [21,25].

**Figure 3:**
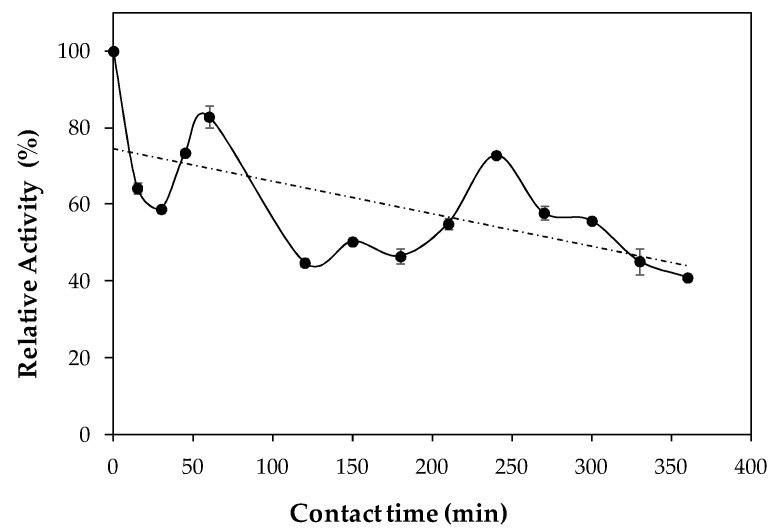
Thermal stability of *Yarrowia lipolytica* lipase immobilized on cell debris (LipImDebri) incubated at 37 °C in 50 mM phosphate buffer pH 7.0. Lines between dots do not represent experimental data.

Thermal stability was tested at 37 °C and pH 7.0, as Figure 3 depicts. A deactivation rate coefficient, k_d_, of 0.08 h^−1^ and a half-life (t_1/2_) of 8.7 h were determined at these conditions. Higher k_d_ and lower t_1/2_ were found for immobilized lipase B from *C. antarctica* obtained by covalent attachment at pH 7.0 [26]. Kumari and Gupta [22] also found lower t_1/2_ for a lipase from *Trichosporon asahii*, which shows that LipImDebri has a good thermal stability at 37 °C.

Among the advantages of immobilized enzyme systems is the possibility of recycling the biocatalyst, which reduces operational costs. Free enzymes are generally soluble and less stable in the reaction medium which requires its replacement in the system [5]. Ota et al. [16] detected, a long time ago, that *Y. lipolytica* cells maintain lipases bound to the cell wall. In the present study, lipase that remained bound to cells after an ultrasonic treatment was tested as a biocatalyst. In this sense, this “natural” immobilization technique reduces cost, since no additional material is used, and time, as immobilization occurs during enzyme production.

**Figure 4:**
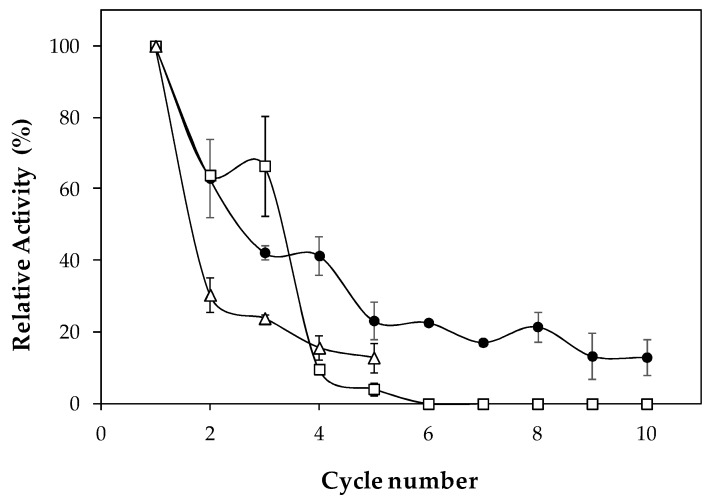
Reuse stability of *Yarrowia lipolytica* lipase immobilized on cell debris (LipImDebri). Reaction conditions: 37 °C, pH 7.0, for 10 min. (•) no washing procedure; (∆) washing LipImDebri with Tris-HCl buffer between cycles; (□) washing LipImDebri with chloroform/methanol (1:1) between cycles. Lines between dots do not represent experimental data.

Figure 4 indicates changes in relative activity of lipase immobilized on cell debris (LipImDebri) as this biocatalyst is recycled. In the second reuse, the relative activity falls to 65% and after the fourth recycle, an even lower activity was detected (40%), reducing to 20% after the eighth cycle. Finally, after ten recycles, the relative activity was marginally 10% of the initial value. Stolarzewicz et al. [27] observed more than 70% decrease in lipase activity in the second use of *Y. lipolytica* biomass with bound lipase, immobilized on alginate beads. Fickers et al. [28] detected LIP7 and LIP8 genes from *Y. lipolytica* which encode two cell-bound lipases that are easily released by washing the cells with phosphate buffer. Since in the present study the cells are treated with ultrasonic waves to obtain the biocatalyst, the lipase that remains attached to cell debris should be strongly bounded, in contrast to the one attached to biomass as reported by Stolarzewicz et al. [27] and therefore, LipImDebri might be a more stable biocatalyst.

Reuse of *C. antarctica* lipase B (CALB) immobilized by physical adsorption on coconut fiber in comparison to CALB immobilized on acrylic resin (commercial lipase—Novozymes 435) showed that the first retained less than 50% of its initial hydrolytic activity after the third cycle, whereas Novozyme 435 could be used with more than 70% of initial activity for ten cycles [29]. The desorption of the enzyme during reaction because of the weak electrostatic interactions between the enzyme and coconut fiber support could be the reason why CALB immobilized on coconut fiber presented lower residual activity. In the present work it is believed that lipase is strongly bound to cell debris. Therefore, a washing step was introduced between cycles to remove any retaining substrate or product, as described by Brigida et al. [29]. The washing procedure with Tris-HCl buffer (pH 7.5) was worse for biocatalyst reuse than without any washing procedure (Figure 4). This washing buffer was tested, and no hydrolytic activity was detected (data not shown), showing that the enzyme is not lixiviating. However, a yellowish color was observed in the washing buffer, demonstrating the presence of the product (*p*-nitrophenol), which might have not been fully removed. Therefore, a combination of miscible and immiscible solvents was initially tested in order to remove hydrophobic or hydrophilic compounds. Indeed, when washing with chloroform/methanol (1:1) solution, a third cycle with remaining activity of above 60% was detected (Figure 4), showing that probably substrate or product was removed from enzyme site. However, in the fourth cycle, the activity dropped drastically, which might be due to the effect of the solvent in the enzyme structure.

Lipases react differently in relation to solvents. Lipase from *Trichosporon asahii* retained most of its residual activity (minimum of 65%) after being in contact for 1 h with water miscible solvents (butanol, isopropanol, acetone, ethanol, and methanol) at 90% (*v*/*v*) and retained 100% of residual activity or more when water immiscible solvents were used (petroleum ether, DMSO, and benzene) [22]. This is in contrast to what has been reported for yeast lipases, which are not generally stable in water miscible solvents [30]. Polar solvents having LogP value less than 2 cause enzyme inactivation because they compete with the enzyme for water molecules. Therefore, water is removed from the enzyme causing protein unfolding, making it unstable [31]. *Y. lipolytica* is known to have 16 different putative lipase genes, out of which *YLIP2* is the main extracellular lipase and most studied one. These lipases show different characteristics, such as lipase 9 from *Y. lipolytica* MSR80 (*ZZ-YLIP9*) that was stable in several solvents (petroleum ether, butanol, hexane, 1,4-dioxane, tetrahydrofuran, acetone, ethanol, and 2-propanol) at 50% (*v*/*v*), tested by Syal and Gupta [32].

**Figure 5:**
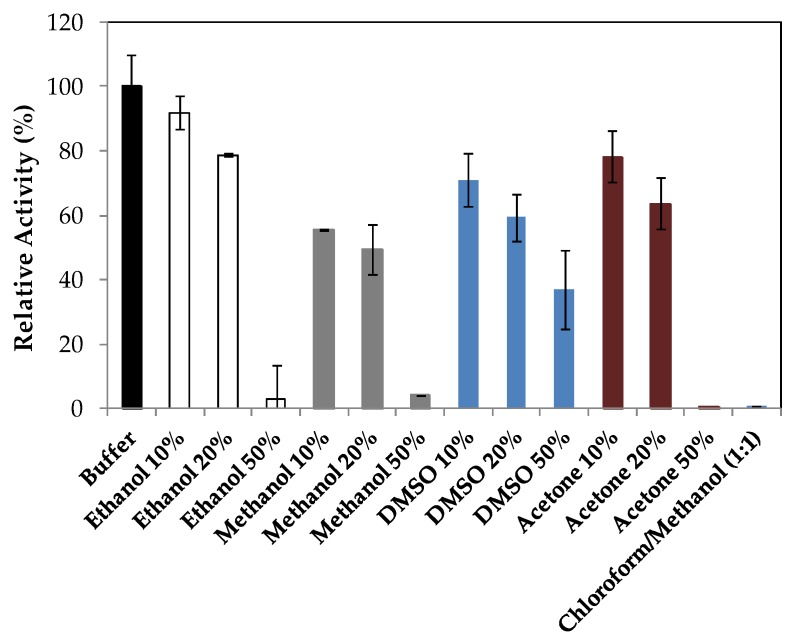
Effect of different solvents (ethanol, methanol, dimethyl sulfoxide DMSO, acetone, and mixture of chloroform/methanol) on the stability of *Yarrowia lipolytica* lipase immobilized on cell debris (LipImDebri) incubated at 37 °C, pH 7.0, for 30 min and assayed for hydrolytic activity in *p*-nitrophenyl laurate. Hydrolytic activity of LipImDebri incubated in phosphate buffer (50 mM, pH 7.0) for 30 min was taken as 100%.

LipImDebri was incubated for 30 min in different solvents with increasing concentrations (Figure 5) and indeed, the mixture of chloroform/methanol (1:1), which was tested as washing solution for reuse, had a drastic effect after a 30 min contact with the enzyme. This might be due to the effect of the polar solvent, methanol (log P = −0.76), that reduced the activity of LipImDebri to almost zero when used at 50% (Figure 5). LipImDebri was more stable in ethanol 10%, but at a concentration of 50%, DMSO was the best solvent.

### 2.3. Anhydrous Milk Fat Production

Anhydrous milk fat was obtained by removing water from nonfermented unsalted butter (produced with bovine milk) and 111.0 g of anhydrous milk fat was obtained with a total yield of 55.5%, inferior than expected since butter generally contains a minimum of 80% fat according to the Codex Alimentarius [33] for traditional milk products. However, the process of removing water did not affect TAG composition as 98.96% TAG was detected for the anhydrous milk fat produced and 99.44% TAG was determined for the butter used in the process. FA composition (g/100 g of total fatty acids) of the milk fat produced (61.8% of saturated FAs and 38.2% of unsaturated FAs—Table 1) was similar to bovine milk fat (69.4% of saturated FAs and 30.6% unsaturated FAs) [34].

Branched-chain FAs were accurately analyzed by a mathematical approach [35], which contributed for a more detailed characterization of milk fat (Table 1). Short-to-medium chain fatty acids (C4:0 to C10:0) represented 2.56% of the total FA content in milk fat, which was rather lower than expected for dairy fats. It should not be discarded the possibility that the partially volatile short-chain fatty acid methyl esters were lost during sample preparation, leading to an underestimation of these fatty acids’ contents. Palmitic acid (16:0) was the major FA in milk fat, which is generally the case for dairy fat [36]. Approximately 32% of the milk fat was composed of monounsaturated fatty acids (MUFAs), mainly oleic acid (18:1*n*-9), which content was slightly higher than in Swedish dairy fat (max. 25%) [34]; this might be due to feeding practices that vary seasonally, since in the summer Swedish milk fat presents higher contents of unsaturated FAs as well. Polyunsaturated fatty acids (PUFAs) represented 3.93% of total FAs and the major PUFAs were linoleic (18:2*n*-6), eicosenoic (20:1*n*-9), γ-linolenic (18:3*n*-6), and α-linolenic acids (18:3*n*-3) (Table 1). Conjugated linoleic acid was also detected in milk fat in similar content to that of Swedish milk fat (0.4% of CLA) [34]. Milk fat is one of most complex natural fats, composed of a wide variety of FAs. This complex lipid mixture is the result of the feed and natural microbial activity in the rumen of the cow; consequently, there is variability in fatty acid composition depending on feed, geographical origin, and climatic factors, among others.

### 2.4. Lipolysis of Milk Fat

Enzymatically modified milk fat can exhibit distinct flavor notes, depending on the extent and specificity of hydrolysis: at a very low extent, lipolysis can impart sensory notes of free-acid richness; at relatively higher extents, the modified fat acquires either a buttery, creamy, or cheesy flavor [18]. LipImDebri, with an average lipolytic activity of 180 U/g d.w., was tested in two amounts (500 and 750 mg) in milk fat hydrolysis reactions. Table 2 shows that when lower lipolytic activity is applied, a gradual increase in FFA/MAG concentration occurs, with the highest product formation detected after 6 h. For the highest amount of LipImDebri used, the highest FA formation was early detected, after 90 min. However, the reaction did not progress until the end (6 h), and the FA contents peaked (19.5%) after 3 h, which were lower than the peak obtained with 500 mg of LipImDebri (28.2%). Taken by the triacylglycerol contents over time it seems that in the case of 750 mg LipImDebri hydrolysis rate was lowered relative to the rate of its synthesis from 4.5 h onwards, as TAG content increased in parallel to decreases in FA or MAG.

The highest degree of hydrolysis obtained by LipImDebri after 6 h (28%) was several-fold higher than observed for commercial *C. lipolytica* lipase (L5TM Amano Pharmaceutical, Nagoya, Japan) in bovine milk fat hydrolysis (5%) reported by Regado et al. [18], that also evaluated this enzyme with goat milk fat (6%) and sheep milk fat (7%).

Three samples of lipolyzed milk fat, herein called LMF, were chosen for further analyses of free fatty acid composition by gas chromatography (GC), after separating the free fatty acid fraction by thin layer chromatography (TLC) (Table 3). For these analyses, LMFs from 4.5 and 6 h of lipolysis with 500 mg LipImDebri were chosen, and to assess the influence of higher amounts of LipImDebri, 750 mg of the catalyst and 3 h reaction duration were also tested. In general, no significant differences (*p* > 0.05) were observed in FA composition between the products of milk fat lipolysis (LMFs).

The major FAs released from milk fat TAG after lipolysis with LipImDebri were oleic acid (18:1*n*-9), palmitic acid (16:0), and stearic acid (18:0) (Table 3). Although these three fatty acids were the major ones in anhydrous milk fat (Table 1), the ranks of the first and second FA were changed, indicating that hydrolysis favored oleic acid over palmitic. These results are consistent with data of this lipase selectivity. Akil et al. [37] have shown that extracellular lipase from *Y. lipolytica* presents typo-selectivity towards long-chain unsaturated FA, especially when compared to saturated fatty acids.

Short chain fatty acids (SFCAs) are important to generate characteristic cheese flavors in combination to other non-lipid substances [18]. The FFA fraction of the LMF obtained with 500 mg of LipImDebri after 6 h of hydrolysis presented 8.13% of odor-active fatty acids (4:0 to 10:0), the highest amount between the LMFs produced, followed by 500 mg of LipImDebri and 4.5 h (6.78%) and 750 mg of LipImDebri and 3 h (6.32%) (Table 3). In this sense, lipolysis time and the amount of enzyme influenced hydrolysis of these short and medium chain FAs from TAGs. Chen and Yang [38] have shown an increase in SCFA release from milk fat when lipase was entrapped in synthetic gel matrices, because of differences in diffusion rates of milk fat TAGs. In the present study, high amounts of odor-active fatty acids were detected in LMF, especially considering that milk fat TAGs presented 2-to-3-fold lower contents of these fatty acids. Extracellular lipase from *Y. lipolytica* has a strictly 1,3-regioselective profile on homogeneous and heterogeneous TAGs [37] and Lubary et al. [39] detected more than 90% of butyric (4:0) and caproic (6:0) acids and more than 70% of caprilyc (8:0) and capric (10:0) acids esterified in the primary positions (*sn*-1 and *sn*-3) of the TAGs in milk fat.

Medium and long chain fatty acids, such as oleic (18:1*n*-9), palmitic (16:0), stearic (18:0), and myristic (14:0) acids were released from milk fat TAGs in high amounts (Table 3). Regado et al. [18] presented similar results in their study in the production of LMF by using industrial lipases from *Pseudomonas fluorescens*, *Candida cylindracea*, *Rhizopus delemar*, and *Candida lipolytica*. Lipase from *Penicillium camembertii* and cutinase from *Fusarium solani pisii* were the best for LMFs with higher amounts of the odor-active SCFAs. The LipImDebri prepared herein showed results comparable or in some cases improved, when compared to commercial lipases in the production of LMF, and merits future scaling-up investigations that should point out if this would be a viable industrial catalyst.

## 3. Materials and Methods

### 3.1. Materials

Bacteriological peptone and yeast extract were purchase from Oxoid (Hampshire, UK). Glucose and agar-agar were supplied by Vetec (Rio de Janeiro, Brazil). Antifoan 204 was obtained from Sigma-Aldrich (St. Louis, MO, USA) to reduce foaming. The Residual Frying Oil (RFO), used as an inductor for lipase production, was supplied by the Brazil Fast Food Corporation, used in Bob’s fast food restaurant (Rio de Janeiro, Brazil) to fry potatoes. Pasteurized nonfermented unsalted butter produced from bovine milk (Bela nata, Laticínios Passa Quatro, Minas Gerais, Brazil) was obtained in local market (Rio de Janeiro, Brazil). Saturated fatty acid ethyl esters (FAEEs) of caprylic (8:0), capric (10:0), lauric (12:0), myristic (14:0), palmitic (16:0), and stearic (18:0) acids, unsaturated FAEEs of oleic (18:1*n*-9), linoleic (18:2*n*-6), and α-linolenic (18:3*n*-3) FAs, triolein standard and fatty acid methyl esters (FAMEs) standards for gas chromatography (GC), all of >99% purity, were purchased from Sigma-Aldrich (Saint Quentin, France).

### 3.2. Strain, Media, and Inoculum Preparation

A wild type strain of *Yarrowia lipolytica* IMUFRJ 50682 was isolated from Baía de Guanabara, Rio de Janeiro, Brazil [40] and was incubated at 4 °C on YPD-agar medium. In the inoculum, cells were cultivated at 28 °C in stirred flasks at 250 rpm, in 500 mL flasks containing 200 mL YPD medium (*w*/*v*: yeast extract 1%; peptone, 2%; glucose, 2%). Cells from the inoculum were centrifuged (26,000× *g*) and used to inoculate 3 L of YPRFO medium (*w*/*v*: yeast Extract 1%; peptone, 2%; residual frying oil 2.5% *v*/*v*) in sufficient amount to obtain 1 g dry weight of cells mL^−1^.

### 3.3. Production of Lipases (Fermentation Process)

Lipase production by *Y. lipolytica* was performed in a 4 L bench bioreator (New Brunswick MF-114, Sci. Inc., USA), containing 3 L of YPRFO medium. Antifoam (2 mL) was added for avoid foaming. Fermentation was performed with three Rushton impellers, with six vertical blades and 4.7 cm diameter, airflow rate of 1.5 L/min, and temperature controlled at 28 °C. These conditions were maintained for 24 h of culture. After this step, the medium was centrifuged (4600× *g*) and cells were used to obtain lipase immobilized in cell debris.

### 3.4. Preparation of Lipase Immobilized in Cell Debris

*Y. lipolytica* cells obtained after the fermentation process were washed with distillated water and 200 mM MOPS (3-morpholinopropane-1-sulfonic acid) buffer pH 7.0 (MERCK, Brazil) and centrifuged at 4 °C, 4600× *g*, for 5 min. Cells were resuspended in MOPS buffer and sonicated in a 20 kHz horn-type sonicator (ultrasonic mixing sonicator, DES500, Unique Group, S.P., Brazil), in an ice water bath, in two stages of constant acoustic power of 150 W and frequency of 20 kHz, for 9 min [15]. After centrifugation (4 °C, 4600× *g*, for 5 min), the sonicated biomass (cell debris with lipase) was resuspended in 200 mM MOPS buffer pH 7.0 and frozen for after measurement of enzyme activity. This sonicated biomass was used as lipase immobilized in cell debris (LipImDebri), as described in Figure 1.

### 3.5. Lipase Activity

Determination of enzymatic activity of lipase immobilized in cell debris (LipImDebri) was performed by hydrolysis of *p*-nitrophenyl laurate (*p*NP-laurate) [15]. In this method 1.9 mL of 560 μM *p*NP-laurate dissolved in 50 mM potassium-phosphate buffer (pH 7.0) containing 1% (*v*/*v*) dimethyl sulfoxide (DMSO) is mixed, at 37 °C, with 0.1 mL of enzyme, containing an amount of LipImDebri determined by dry weigh (d.w.) measurement. For extracellular and intracellular lipase activity, 0.1 mL of those crude extracts were used. The production of *p*-nitrophenol (product of the enzymatic reaction) is followed by 100 s in a spectrophotometer (HACH, DR/4000U) at λ = 410 nm (extinction coefficient under these conditions is 10.052 × 1/mol/cm). One lipase unit (U) is defined as the amount of enzyme which releases 1 μmol of *p*-nitrophenol per minute at pH 7.0 and 37 °C.

### 3.6. Effect of Temperature and pH on Lipase Immobilized in Cell Debris

The activity of the biocatalyst (LipImDebri) in the hydrolysis of *p*NP-laurate was determined in the range 25 to 75 °C in 50 mM potassium-phosphate buffer, pH 7.0, as described in Section 3.5. The activity was also determined at different pH ranging from 4.0 to 10.0 using 50 mM of each buffer, including acetate buffer (pH 4–5), potassium phosphate buffer (pH 6–7) and Tris-HCl buffer (pH 8–10), at 37 °C, as described in Section 3.5.

### 3.7. Thermal Stability and Stability in Different Solvents of Lipase Immobilized in Cell Debris

Thermal stability was determined by incubating the biocatalyst (LipImDebri) in 50 mM potassium phosphate buffer at 37 °C and pH 7.0. Samples were withdrawn at different times for 6 h and their residual activities were assayed by hydrolysis of *p*NP-laurate as described in Section 3.5. Hydrolytic activity before incubation was taken as 100%. First-order deactivation rate coefficient (k_d_) was estimated from experimental data, using Equation (1) [26]:

ln(LA) = −k_d_ × t + ln(LA_i_) (1)

where LA_i_ is the initial residual lipase activity, LA is the residual lipase activity in time t, and k_d_ is first-order deactivation rate coefficient. The biocatalyst half-life (t_1/2_) was estimated by Equation (2) using the estimated parameter k_d_ [26].

t_1/2_ = −ln(0.5)/(−k_d_) (2)

The activity of the biocatalyst (LipImDebri) was assayed by the method described in Section 3.5 after 30 min incubation at 37 °C in different solvents (ethanol, acetone, dimethyl sulfoxide (DMSO), and acetone) at different concentration (10%, 20%, and 50%) as well as in pure chloroform/methanol (1:1). Hydrolytic activity of LipImDebri incubated in phosphate buffer (50 mM, pH 7.0) for 30 min was taken as 100%.

### 3.8. Repeated Use of Lipase Immobilized in Cell Debris

To verify the reuse of the biocatalyst (LipImDebri) consecutive hydrolysis of *p*NP-laurate 560 μM, as described in Section 3.5, was performed. After each hydrolysis reaction, LipImDebri was centrifuged at 4 °C, 4600× *g*, the supernatant was discarded and the biocatalyst was used again in hydrolysis reaction. This process was performed 10 times. A washing procedure between cycles was also tested with 50 mM Tris_HCl buffer (pH 7.5) and with chloroform/methanol (1:1) solution. After washing, LipImDebri was again centrifuged at 4 °C, 4600× *g*, the supernatant was discarded and the reaction medium (*p*NP-laurate 560 μM) replaced.

### 3.9. Preparation of Anhydrous Milk Fat

Pasteurized unsalted nonfermented butter was pretreated according Kalo et al. [41] with some modifications [42]. Water was removed using a separating funnel at 60 °C and fat was filtered in normal paper filter and dried in vacuum condition 800 mbar for 1 h in a boiling water bath. Anhydrous nitrogen was bubbled in the melted butterfat for 5 min to remove the residual oxygen and water. The dried milk fat was stored in 500 mL Scott flasks at −30 °C until characterization and hydrolysis reactions.

### 3.10. Milk Fat Hydrolysis Reactions

The hydrolysis reactions of milk fat were carried out at 37 °C, following a protocol adapted from Regado et al. [18]. Anhydrous milk fat stored in Scott flasks was heated at 40 °C in water bath. This milk fat (0.5 mL) was transferred to an amber flask, where 15 mL of 50 mM of Tris-HCl buffer (pH 7.0) and enzyme (LipImDebri) were added. The reaction was performed in rotary incubator at 37 °C and at 250 rpm. The following reaction conditions were evaluated: amount of LipImDebri (0.50, 0.75, and 1.00 g) and hydrolysis time (1.5, 3.0, 4.5, and 6 h).

### 3.11. Monitoring Lipid Composition during Milk Fat Hydrolysis Reactions

#### 3.11.1. High Performance Liquid Chromatography (HPLC)

Milk fat hydrolysis was monitored by determination of lipid classes following a procedure adapted from Tan and Brzuskiewicz [43] in a HPLC (Pump LC-20at, Degasser DGU-20A5 and communicator module CBM-20A; Shimadzu^®^, Japan) (Figure S1). Samples were dissolved in an injection solution, composed of acetonitrile:isopropanol:hexane (2:2:1, *v*/*v*/*v*) and a reversed-phase HPLC column (C18, 5 µm, 250 mm × 4.6 mm, Kromasil^®^; AkzoNobel^®^, Sweden) was used. The lipid analytes were eluted with a mobile phase gradient of acetonitrile (A) and isopropanol (B), at 1.0 mL/min, from 0 to 69% B from 0 to 60 min, followed by re-equilibration until 0% B, from 60 to 76 min (Table S1). The eluate was monitored with an evaporative light scattering detector (ELSD-LT II; Shimadzu^®^, Japan), using N_2_ as nebulizer gas at 0.65 mL/min, and drift tube temperature at 40 °C. Lipid classes of TAGs-DAGs and FFAs-MAGs were identified comparing with commercial standards and quantified by internal normalization. Results were expressed in g/100 g of total lipids.

#### 3.11.2. Thin Layer Chromatography (TLC)

After considering the results from HPLC analysis, the three reaction conditions with the highest three percentages of FFA and MAG were selected for lipid class fractionation by TLC following a procedure adapted from Akil et al. [37]. Samples were extracted in *n*-hexane, dried under a gentle stream of nitrogen gas and separated by TLC. For each analysis, 90 µL of the *n*-hexane extract (2 mg/mL) was applied on aluminum TLC silica gel Plate G60 with concentration zone (Merck, Darmstadt, Germany) using a volumetric microsyringe. Lipid classes were separated using *n*-hexane:diethyl ether:formic acid (70:30:1, *v*/*v*/*v*) solution as mobile phase in a TLC glass tank. The lane containing a standards mixture solution, containing TAG, diacylglycerol (DAG), MAG, and FFA that was run in parallel on the TLC plate was revealed with a copper sulfate (saturated):phosphoric acid:methanol:water (50:40:25:390, *v*/*v*/*v*/*v*) revelation solution, and chromatographic bands migration was used to identify lipid classes in samples. Thin layer chromatography plates were carefully cut for revelation of lipids standards by heating at 180 °C for 7 min. The area in the plate representing FFA band was scraped-off, and the FAs in this TLC bands were analyzed by gas chromatography (GC) after proper derivatization.

#### 3.11.3. Analysis of Fatty Acid Composition by Gas Chromatography (GC)

The bands of free fatty acids scraped from the TLC plates were analyzed by gas chromatography (Figure S2). The free fatty acids were methylated according to Lepage & Roy [44]. A GC-2010 gas-chromatograph (Shimadzu, Japan) was used for all analyzes, and the split/splitless injector was operated with a 1:30 split ratio. A moderately polar capillary column (polyethylene glycol; Omegawax-320, 30 m, 0.32 mm i.d., 0.25 μm film thickness; Sigma-Aldrich, São Paulo, Brazil) was used to separate the fatty acid methyl esters, using He as carrier gas (25.0 cm/s). The injector and detector temperatures were set at 260 and 280 °C, respectively. Column oven temperature was held at 40 °C for 3 min, then temperature programmed at 6.5 °C/min to 180 °C and held for 3 min, then temperature programmed at 2.0 °C/min to 210 °C and held for 15 min (Table S1). Identification of fatty acid methyl esters (FAME) was made by comparison to relative retention times of commercial standards. The Equivalent Chain Lengths [45] and the mathematical method described by Torres et al. [35] were used to complement the characterization of samples’ FAs, through analysis of FAME peaks not present in the standard mixture. The fatty acids were expressed as mol% of total fatty acids. All analyses were performed in duplicates.

### 3.12. Statistical Analysis

Analysis of variance (ANOVA) was used to compare the results and a post-hoc test was conducted to determine differences among mean values using Turkey HSD. Homogeneity of variance was verified by Levene test. Means with *p* < 0.05 were considered significantly different.

## 4. Conclusions

Residual frying oil from a fast food restaurant was considered an efficient inducer for extracellular, intracellular, and cell wall-bound lipase production during *Y. lipolytica* growth in a 4 L bioreactor. Additionally, the lipase that remained attached to cell debris after sonication (LipImDebri) showed a potential to be used as an immobilized enzyme that could be reused for four times with more than 40% residual hydrolytic activity. LipImDebri was able to produce lipolyzed milk fat (LMF) from anhydrous milk fat after 6 h of lipolysis with high amounts of palmitic, oleic, and stearic acids (major acids), but also high amounts of the odor-active short-to-medium chain fatty acids (8%). The hydrolysis of milk fat with lipase immobilized in cell debris can be used in the dairy industry to produce flavor compounds to be added in foods. The reduced production and immobilization costs due to the use of low-cost raw material (RFO) and the microbial cell used as support material makes LipImDebri an interesting biocatalyst for the food industry, especially for hydrolysis reactions.

## Figures and Tables

**Table 1:** Fatty acid composition (mol%) of anhydrous milk fat.

Fatty Acids	Contents (%, mol/mol)	Fatty Acids	Contents (%, mol/mol)
**Saturated**		**Monounsaturated**	
4:0	0.63 ± 0.45	14:1	0.66 ± 0.02
6:0	0.48 ± 0.23	15:1	0.28 ± 0.01
8:0	1.35 ± 0.33	16:1	1.30 ± 0.06
10:0	0.11 ± 0.03	17:1	0.84 ± 0.01
11:0	0.16 ± 0.06	18:1*n*-9	28.5 ± 2.03
12:0	1.88 ± 0.17	**Polyunsaturated**	
13:0	0.06 ± 0.00	18:2*n*-6	2.17 ± 0.03
14:0	9.13 ± 0.26	18:3*n*-6	0.43 ± 0.02
15:0	0.94 ± 0.03	18:3*n*-3	0.44 ± 0.02
16:0	31.9 ± 0.79	20:1*n*-9	0.75 ± 0.02
17:0	0.50 ± 0.01	20:3*n*-6	0.03 ± 0.00
18:0	16.0 ± 0.15	20:3*n*-3	0.10 ± 0.01
20:0	0.24 ± 0.01	**Conjugated**	
21:0	0.06 ± 0.01	CLA *	0.35 ± 0.02
22:0	0.12 ± 0.02		
**Branched-chain**			
i14:0	0.08 ± 0.00		
i15:0	0.26 ± 0.01		
i18:0	0.29 ± 0.00		

Results are expressed as mean ± standard deviation of replicates. * CLA: conjugated linoleic acid (18:2); tentatively identified as *cis*, *trans* isomer group.

**Table 2:** Lipid profile relative to milk fat lipolysis with *Y. lipolytica* lipase immobilized in cell debris (LipImDebri).

Time of Lipolysis (h)	Lipid Classes	
	500 mg LipImDebri	750 mg LipImDebri
	Free Fatty Acid (FFA) + Monoacylglycerol (MAG) Fraction (%)	
0	0.00 ^a,C^	0,00 ^a,C^
1.5	0.98 ± 0.29 ^a,C^	5.48 ± 2.53 ^a,B,C^
3.0	11.02 ± 1.00 ^a,B,C^	19.54 ±0.94 ^b,A,B,C^
4.5	17.86 ± 5.84 ^a,A,B^	11.79 ± 4.58 ^a,A,B^
6.0	28.23 ± 3.45 ^a,A^	14.30 ± 5.34 ^a,A,B^
	**Diacylglycerol (DAG) Fraction (%)**	
0	1.11 ± 0.02 ^a,A^	1.11 ± 0.02 ^a,A^
1.5	0.31 ± 0.34 ^a,A^	0.57 ± 0.15 ^a,A^
3.0	1.57 ± 0.94 ^a,A^	1.27 ± 0.02 ^a,A^
4.5	1.18 ± 0.52 ^a,A^	0.62 ± 0.20 ^a,A^
6.0	0.56 ± 0.05 ^a,A^	1.41 ± 0.48 ^a,A^
	**Triacylglycerol (TAG) Fraction (%)**	
0	98.96 ± 0.11 ^a,A^	98.96 ± 0.11 ^a,A^
1.5	98.71 ± 0.63 ^a,A^	93.95 ± 2.68 ^a,A,B^
3.0	87.41 ± 1.95 ^a,B^	79.19 ± 0.96 ^b,C^
4.5	80.96 ± 5.30 ^a,B^	87.60 ±4.37 ^a,AB,C^
6.0	72.71 ± 5.52 ^a,C^	84.29 ± 4.86 ^a,B,C^

Results are expressed as mean ± standard deviation of replicates. Nonhydrolyzed milk fat was considered the time zero (without hydrolysis). Statistical analyses (FFA + MAG, DAG, and TAG were considered independently): different letters in the same row (lower case) and in the same column (upper case) indicate significant differences (ANOVA; *p* < 0.05).

**Table 3:** Fatty acid composition (mol%) of the free fatty acids fraction produced by lipolysis of milk fat by *Y. lipolytica* lipase immobilized in cell debris (LipImDebri).

Fatty Acid	LipImDebri (mg), Lipolysis Time (h)		
	500, 4.5	500, 6	750, 3
**Saturated**			
4:0	0.74 ± 0.29 ^a,D^	0.51 ± 0.13 ^a,D^	0.76 ± 0.53 ^a,E^
6:0	0.45 ± 0.09 ^a,D^	0.27 ± 0.02 ^a,D^	0.65 ± 0.49 ^a,E^
8:0	4.40 ± 1.98 ^a,C,D^	6.79 ± 5.30 ^a,D^	3.63 ± 3.46 ^a,E,F^
10:0	0.74 ± 0.02 ^a,C,D^	0.56 ± 0.15 ^a,D^	1.44 ± 1.16 ^a,E^
12:0	1.99 ± 0.83 ^a,C,D^	2.42 ± 1.57 ^a,D^	2.74 ± 0.16 ^a,D,E^
14:0	8.17 ± 0.03 ^a,C^	6.03 ± 0.99 ^a,D^	8.14 ± 2.20 ^a,D^
15:0	0.90 ± 0.05 ^a,C,D^	0.79 ± 0.12 ^a,D^	0.81 ± 0.01 ^a,E^
16:0	28.80 ± 3.00 ^a,A^	24.97 ± 6.94 ^a,B^	24.67 ± 0.71 ^a,B^
18:0	13.94 ± 1.60 ^a,B^	15.36 ± 3.25 ^a,C^	12.79 ± 1.60 ^a,C^
**Branched-Chain**			
i15:0	0.22 ± 0.01 ^a,D^	0.22 ± 0.01 ^a,D^	0.21 ± 0.03 ^a,E^
a15:0	0.34 ± 0.03 ^a,C,D^	0.29 ± 0.05 ^a,D^	0.31 ± 0.06 ^a,E^
**Monounsaturated**			
14:1	0.56 ± 0.09 ^a,C,D^	0.40 ± 0.06 ^a,D^	0.69 ± 0.41 ^a,E^
16:1	1.19 ± 0.27 ^a,C,D^	1.07 ± 0.08 ^a,D^	1.32 ± 0.39 ^a,E^
17:1	0.69 ± 0.13 ^a,C,D^	0.64 ± 0.02 ^a,D^	0.56 ± 0.08 ^a,E^
18:1*n*-9	31.27 ± 5.16 ^a,A^	35.18 ± 1.83 ^a,A^	35.31 ± 1.05 ^a,A^
**Polyunsaturated**			
18:2*n*-6	4.29 ± 1.24 ^a,C,D^	3.84 ± 0.54 ^a,D^	4.82 ± 0.47 ^a,D,E^
20:1*n*-9	1.33 ± 0.32 ^a,C,D^	0.67 ± 0.07 ^a,D^	0.85 ± 0.18 ^a,E^

Results are expressed as mean ± standard deviation of replicates. Statistical analysis: different letters in the same row (lower case) and in the same column (upper case) indicate significant differences (*p* < 0.05).

## Supplementary Materials

Supplementary materials can be found at http://www.mdpi.com/1422-0067/19/11/3413/s1.

## Abbreviations

HPLC	High-performance liquid chromatography
GC	Gas chromatography
TLC	Thin layer chromatography
LMF	Lipolyzed milk fat
LipImDebri	Lipase immobilized in cell debris

## References

[1] Cao Z.M., Li J., Wang W., Ge T., Yue R., Li V.L., Colvin W., Yu W. (2012). Food related applications of magnetic iron oxide nanoparticles: Enzyme immobilization, protein purification, and food analysis. Trends Food Sci. Technol..

[2] Kumar A., Venkatesu P. (2002). Overview of the stability of α-chymotrypsin in different solvent media. Chem. Rev..

[3] Verma N., Thakur S., Bhatt A.K. (2012). Microbial lipases: Industrial applications and properties (a review). Int. Res. J. Biol. Sci..

[4] de Oliveira U.M., de Matos L.J.L., de Souza M.C.M., Pinheiro B.B., dos Santos J.C., Gonçalves L.R. (2018). Effect of the presence of surfactants and immobilization conditions on catalysts’ properties of *Rhizomucor miehei* lipase onto chitosan. Appl. Biochem. Biotechnol..

[5] Mateo C., Palomo J.M., Fernandez-Lorente G., Guisan J.M., Fernandez-Lafuente R. (2007). Improvement of enzyme activity, stability and selectivity via immobilization techniques. Enzym. Microb. Technol..

[6] DiCosimo R., McAuliffe J., Poulose A.J., Bohlmann G. (2013). Industrial use of immobilized enzymes. Chem. Soc. Rev..

[7] Galvão W.S., Pinheiro B.B., Golçalves L.R.B., de Mattos M.C., Fonseca T.S., Regis T., Zampieri D., dos Santos J.C.S., Costa L.S., Correa M.A. (2018). Novel nanohybrid biocatalyst: Application in the kinetic resolution of secondary alcohols. J. Mater. Sci..

[8] Zdarta J., Meyer A.S., Jesionowski T., Pinelo M. (2018). Developments in support materials for immobilization of oxidoreductases: A comprehensive review. Adv. Colloid Interface Sci..

[9] Sheldon R.A., van Pelt S. (2013). Enzyme immobilisation in biocatalysis: Why, what and how. Chem. Soc. Rev..

[10] Cantone S., Ferrario V., Corici L., Ebert C., Fattor D., Spizzo P., Gardossi L. (2013). Efficient immobilisation of industrial biocatalysts: Criteria and constraints for the selection of organic polymeric carriers and immobilisation methods. Chem. Soc. Rev..

[11] Fukuda H., Hama S., Tamalampudi S., Noda H. (2008). Whole-cell biocatalysts for biodiesel fuel production. Trends Biotechnol..

[12] Adamczak M., Bednarski W. (2004). Enhanced activity of intracellular lipases from *Rhizomucor miehei* and *Yarrowia lipolytica* by immobilization on biomass support particles. Process Biochem..

[13] Barth G., Gaillardin C. (1977). Physiology and genetics of the dimorphic fungus *Yarrowia lipolytica*. FEMS Microbiol. Rev..

[14] Fickers P., Nicaud J.M., Gaillardin C., Destain J., Thonart P. (2004). Carbon and nitrogen sources modulate lipase production in the yeast *Yarrowia lipolytica*. J. Appl. Microbiol..

[15] Nunes P., Martins A.B., Santa Brigida A.I., Miguez Da Rocha Leao M.H., Amaral P. (2014). Intracellular lipase production by *Yarrowia lipolytica* using different carbon sources. Chem. Eng. Trans..

[16] Ota Y., Gomi K., Kato S., Sugiura T., Minoda Y. (1982). Purification and some properties of cell-bond lipase from *Saccharomycopsis lipolytica*. Agric. Biol. Chem..

[17] Kilcawley K.N. (2001). The enzyme effect. Dairy Ind. Int..

[18] Regado M.A., Cristóvão B.M., Moutinho C.G., Balcão V.M., Aires-Barros R., Ferreira J.P.M., Malcata F.X. (2007). Lipolysis of milkfats for flavour. Int. J. Food Sci. Technol..

[19] Pereira A.S., Fraga J.L., Diniz M.M., Fontes-Sant’Ana G.F., Amaral P.F.F. High catalytic activity of lipase from *Yarrowia lipolytica* immobilized by microencapsulation. Int. J. Mol. Sci..

[20] Choe E., Min D.B. (2007). Chemistry of deep-fat frying oils. J. Food Sci..

[21] Yu M., Qin S., Tan T. (2007). Purification and characterization of the extracellular lipase Lip2 from *Yarrowia lipolytica*. Process Biochem..

[22] Kumari A., Gupta R. (2012). Purification and Biochemical Characterization of a Novel Magnesium Dependent Lipase from *Trichosporon asahii* MSR 54 and its Application in Biodiesel Production. Asian J. Biotechnol..

[23] Brígida A.I., Amaral P.F.F., Gonçalves L.R., Rocha-Leão M.H.M., Coelho M.A.Z. (2014). *Yarrowia lipolytica* IMUFRJ 50682: Lipase production in a multiphase bioreactor. Curr. Biochem. Eng..

[24] Kumari A., Verma V.V., Gupta R. (2012). Comparative biochemical characterization and in silico analysis of novel lipases Lip11 and Lip12 with Lip2 from *Yarrowia lipolytica*. World J. Microbiol. Biotechnol..

[25] Castro-Ochoa L.D., Rodríguez-Gómez C., Valerio-Alfaro G., Ros R.O. (2005). Screening, purification and characterization of the thermoalkalophilic lipase produced by *Bacillus thermoleovorans* CCR11. Enzym. Microb. Technol..

[26] Brígida A.I., Pinheiro Á.D., Ferreira A.L., Pinto G.A., Gonçalves L.R. (2007). Immobilization of *Candida antarctica* lipase B by covalent attachment to green coconut fiber. Appl. Biochem. Biotecnol..

[27] Stolarzewicz I.A., Zaborniak P., Fabiszewska A.U., Białecka-Florjańczyka E. (2017). Study on the Properties of Immobilized Biocatalysts with Lipase Activity Produced by *Yarrowia lipolytica* in Batch Culture. Chem. Biochem. Eng. Q..

[28] Fickers P., Fudalej F., Nicaud J., Destain J.E., Thonart P. (2005). Selection of new over-producing derivates for the improvement of extracellular lipase production by the nonconventional yeast *Yarrowia lipolytica*. J. Appl. Microb..

[29] Brígida A.I., Pinheiro Á.D., Ferreira A.L., Gonçalves L.R. (2007). Immobilization of *Candida antarctica* lipase B by adsorption to green coconut fiber. Biotech. Fuels Chem..

[30] Kakugawa K., Shobayashi M., Suzuki O., Miyakawa T. (2002). Purification and characterization of a lipase from the glycolipid-producing yeast *Kurtzmanomyces* sp. I-11. Biosci. Biotechnol. Biochem..

[31] Balan A., Ibrahim D., Rahim R.A. (2013). Organic-solvent and surfactant tolerant thermostable lipase, isolated from a thermophilic bacterium, *Geobacillus thermodenitrificans* IBRL-NRA. Adv. Stud. Biol..

[32] Syal P., Gupta R. (2015). Cloning, expression, and biochemical characterization of an enantioselective lipase, *YLIP9*, from *Yarrowia lipolytica* MSR80. Appl. Biochem. Biotechnol..

[33] FAO, WHO (2007). Standard for Butter. Codex Stan 279, Revised 1999, Amended 2003 and 2006.

[34] Månsson H.L. (2008). Fatty acids in bovine milk fat. Food Nutr. Res..

[35] Torres A.G., Trugo N.M., Trugo L.C. (2002). Mathematical method for the prediction of retention times of fatty acid methyl esters in temperature-programmed capillary gas chromatography. J. Agric. Food Chem..

[36] Lindmark-Månsson H., Fondén R., Pettersson H.E. (2003). Composition of Swedish dairy milk. Int. Dairy J..

[37] Akil E., Carvalho T., Bárea B., Finotelli P., Lecomte J., Torres A.G., Amaral P., Villeneuve P. (2016). Accessing regio-and typo-selectivity of *Yarrowia lipolytica* lipase in its free form and immobilized onto magnetic nanoparticles. Biochem. Eng. J..

[38] Chen J.Y.P., Yang B.A. (1992). Enhancement of release of short-chain fatty acids from milk fat with immobilized microbial lipase. J. Food Sci..

[39] Lubary M., Hofland G.W., Ter Horst J.H. (2011). The potential of milk fat for the synthesis of valuable derivatives. Eur. Food Res. Technol..

[40] Haegler A.N., Mendonça Haegler L.C. (1981). Yeast from marine and estuarine waters with different levels of pollution in the State of Rio de Janeiro, Brazil. Appl. Environ. Microbiol..

[41] Kalo P., Huotari H., Antila M. (1990). *Pseudomonas fluorescens* lipase-catalysed interesterification of butterfat in the absence of a solvent. Milchwiss. Milk Sci. Int..

[42] Balcão V.M., Malcata F.X. (1997). Lipase-catalyzed modification of butterfat via acidolysis with oleic acid. J. Mol. Catal. B Enzym..

[43] Tan B., Brzuskiewicz L. (1989). Separation of tocopherol and tocotrienol isomers using normal- and reverse-phase liquid chromatography. Anal. Biochem..

[44] Lepage G., Roy C.C. (1986). Direct transterification of all classes of lipids in a one-step reaction. J. Lipid Res..

[45] Mhøs S., Grahl-Nielsen O. (2006). Prediction of gas chromatographic retention of polyunsaturated fatty acid methyl esters. J. Chromatogr. A.

