# Supplementary material for: Use of Yarrowia lipolytica Lipase Immobilized in Cell Debris for the Production of Lipolyzed Milk Fat (LMF)
Source: Int J Mol Sci. 2018 Oct 31;19(11):3413. doi: 10.3390/ijms19113413 (PMC6274823; doi:10.3390/ijms19113413)
Supplement: Supplementary file 1 [file ijms-19-03413-s001.zip › Fraga_etal_ Figure S1.pdf]

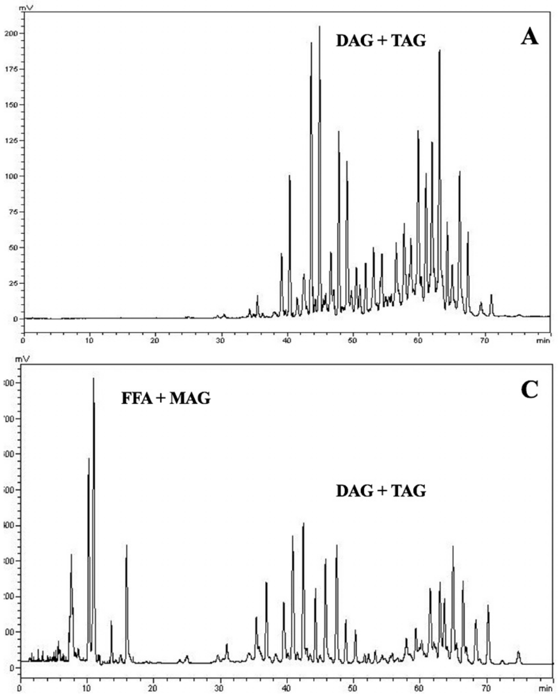

Figure S1. Representative chromatograms of lipid classes analysis by reversed-phase high performance liquid chromatography (HPLC) showing lipolysis of milk fat by *Yarrowia lipolytica* lipase immobilized on cell debris (LipImDebri). FFA + MAG, free fatty acids + monoacylglycerol classes; DAG + TAG, diacylglycerol + triacylglycerol classes. **A**: non-hydrolyzed milk fat; **B**: hydrolyzed milk fat by 500 mg of LipImDebri for 4.5 h; **C:** hydrolyzed milk fat by 500 mg of LipImDebri for 6 h; **D:** hydrolyzed milk fat by 750 mg of LipImDebri for 3 h.
