# Supplementary material for: Use of Yarrowia lipolytica Lipase Immobilized in Cell Debris for the Production of Lipolyzed Milk Fat (LMF)
Source: Int J Mol Sci. 2018 Oct 31;19(11):3413. doi: 10.3390/ijms19113413 (PMC6274823; doi:10.3390/ijms19113413)
Supplement: Supplementary file 1 [file ijms-19-03413-s001.zip › Fraga_etal_Figure S2.pdf]

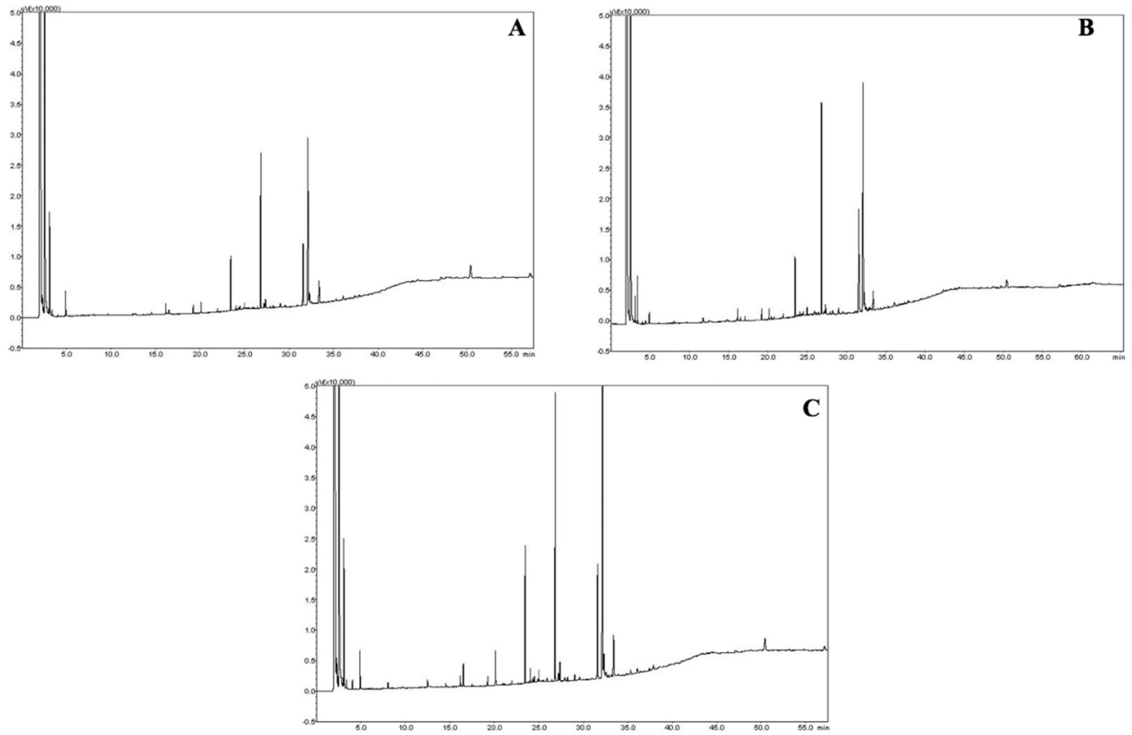

Figure S2. Representative chromatograms for the analysis of fatty acid methyl esters by gas chromatography (GC). The fatty acid profile is from the free fatty acids fraction after milk fat lipolysis by *Yarrowia lipolytica* lipase immobilized with cell debris (LipImDebri). The free fatty acids fraction was isolated by thin layer chromatography on silica-gel plates and the bands corresponding to FFA was scraped-off before methylation. **A**: hydrolyzed milk fat by 500 mg of LipImDebri for 4.5 h; **B:** hydrolyzed milk fat by 500 mg of LipImDebri for 6 h; **C:** hydrolyzed milk fat by 750 mg of LipImDebri for 3 h.
