# Supplementary material for: Use of Yarrowia lipolytica Lipase Immobilized in Cell Debris for the Production of Lipolyzed Milk Fat (LMF)
Source: Int J Mol Sci. 2018 Oct 31;19(11):3413. doi: 10.3390/ijms19113413 (PMC6274823; doi:10.3390/ijms19113413)
Supplement: Supplementary file 1 [file ijms-19-03413-s001.zip › Fraga_etal_Table S1.pdf]

Table S1. High performance liquid chromatography (HPLC) solvent gradient and gas chromatography (GC) temperature gradient, used for analysis of lipid classes, and fatty acid methyl esters, respectively.

Time (min)	HPLC solvents gradient (%)	
	Acetonitrile	Isopropanol
0	100	0
60	31	69
61	100	0
76	100	0
**Time (min)**	**GC temperature gradient (°C)**	
	**Column oven (°C)**	**Rate (°C/min)**
0 to 3	40	-
3 to 25	gradient	6.5
25 to 28	180	
28 to 43	gradient	2.0
43 to 58	210	
